# The Pisciarelli main fumarole mechanisms reconstructed by electrical resistivity and induced polarization imaging

**DOI:** 10.1038/s41598-021-97413-1

**Published:** 2021-09-20

**Authors:** A. Troiano, R. Isaia, F. D. A. Tramparulo, M. G. Di Giuseppe

**Affiliations:** Istituto Nazionale di Geofisica e Vulcanologia, Sezione di Napoli ‘Osservatorio Vesuviano’, Naples, Italy

**Keywords:** Geophysics, Volcanology

## Abstract

Pisciarelli, together with the adjacent Solfatara maar-diatreme, represents the most active structure of the Campi Flegrei caldera (Italy) in terms of degassing and seismic activity. This paper aims to define the structure of the Pisciarelli hydrothermal system (down to a 20 m depth) through electrical resistivity and time-domain-induced polarization tomography and self-potential mapping. The retrieved 3D image of the area helps reconstruct the Pisciarelli subsurface in its area of maximum degassing, containing the main fumarole (“soffione”) and the mud pool. In particular, a channel has been identified in which fluids stored in a deeper reservoir rise toward the surface. Such a structure seems to be surmounted by a clay-cap formation that could govern the circulation of fluids and the abundance of gases/vapors emitted by the soffione. Based on this new reconstruction of the Pisciarelli fumarolic field structural setting, the first conceptual model has been suggested that is capable of simultaneously explaining the mechanisms governing soffione activity and elucidating the role played by the fluid/gas of deeper origin in the shallow fluid circulation system. The proposed model can potentially help to better monitor the processes occurring throughout the Pisciarelli fumarolic field and provide an evaluation of the associated hazards.

## Introduction

**Figure 1:**
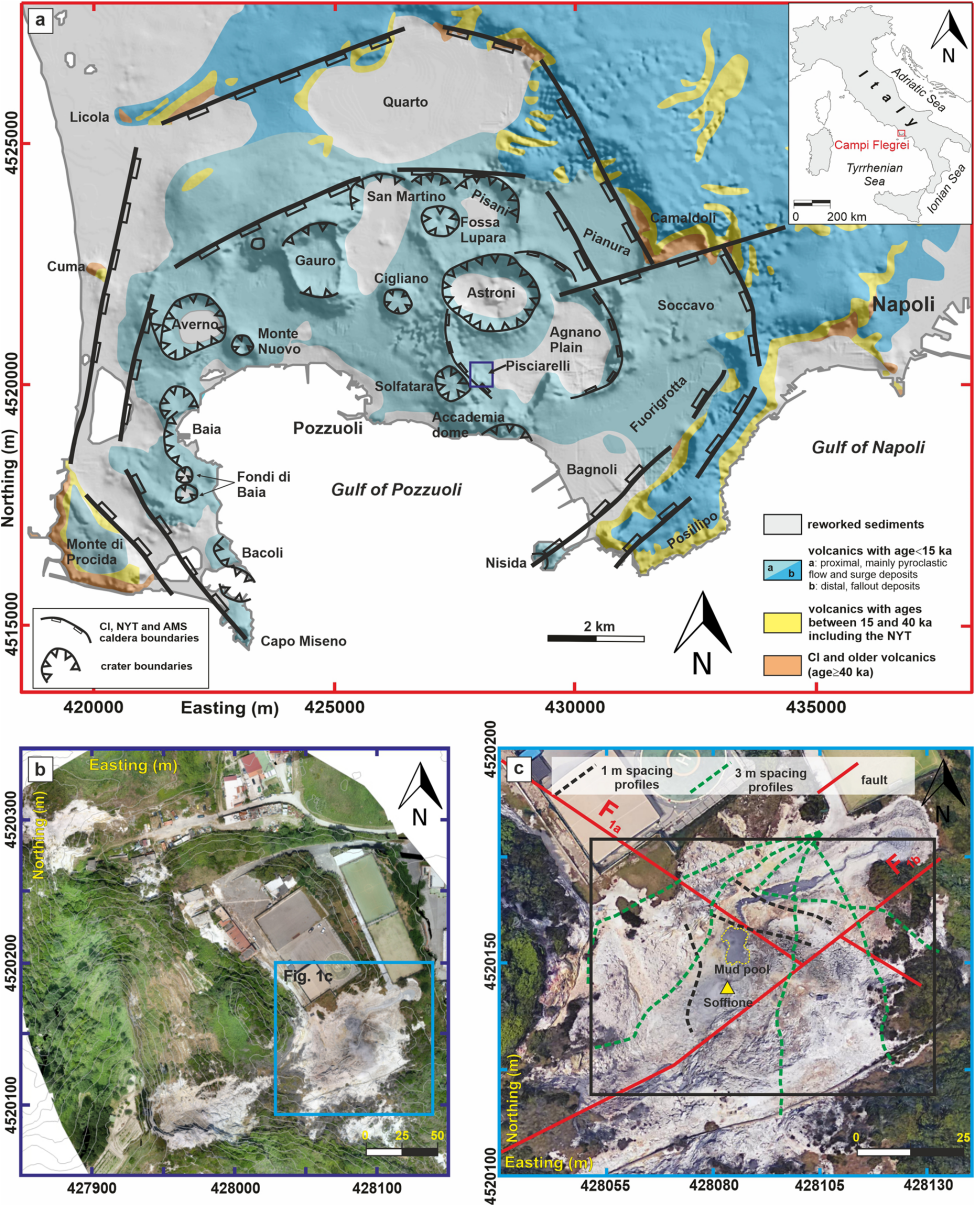
(**a**) Map of the Campi Flegrei caldera (redrawn from ref.^18^). The blue box delineates the location of the PFF site. (**b**) Aerial view of the PFF. The cyan box shows the study area where the high-resolution ERT and TDIP surveys were carried out. (**c**) Aerial view of the study area in the PFF. The solid red lines indicate the two main fault systems identified by ref.^9^. Green and black  dotted lines indicate the locations of the profiles. The yellow triangle indicates the location of the main vent (“Soffione”) (UTM projections, zone 33, datum WGS84, m). The figure was created using the MATLAB R2020b commercial software (https://www.mathworks.com/products/MATLAB.html) and postprocessed using the Corel Draw 17 commercial software (https://www.coreldraw.com/it/product/coreldraw/). The aerial view of Pisciarelli was produced by the Istituto Nazionale di Geofisica e Vulcanologia (INGV), Sezione di Napoli ‘Osservatorio Vesuviano’ (Curtesy of dr. Enrica Marotta).

The most relevant signs that the hydrothermally active Campi Flegrei volcanic field (CF; Fig. 1a) is reawakening are, for example, the intense increases in surface uplift^1^, degassing^2^, and seismicity^3,4^. The hydrothermal area of Pisciarelli (Fig. 1b), together with the adjacent Solfatara maar-diatreme^5,6^, currently represents the most active structure on the caldera in terms of degassing and seismic activity^3^, additionally manifesting significant morphological variations, changes in the geochemical characteristics of the gases/fluids, the opening of new fumarolic vents, the occurrence of several seismic events, and some episodes of mud emission that have been recorded during the last decade^3,7–9^. Moreover, urbanization around the site is extremely high, which enhances the relevance of Pisciarelli in the actual unrest associated with the CF. Nevertheless, few studies have investigated this sector of the CF. In recent years, measurements of the gas flux from hydrothermal sites based on the in situ and remote sensing determination of CO_2_ have been developed in Solfatara and Pisciarelli^10–14^. Concerning the specificity of Pisciarelli, a geochemical investigation recently identified a clear escalation of degassing activity since 2012, with the main fumarole (hereafter indicated as a “soffione”) releasing > 600 tons/day of CO_2_, equivalent to the daily discharge of a medium-size arc volcano approaching an eruptive phase^15^. Furthermore, studies conducted on the seismic tremor generated by fumarolic emissions showed a threefold increase over the last 2 years^16^, suggesting that such an escalation could be driven by an increase in gas transport and venting^17^. In addition, digital photogrammetry conducted through unmanned aerial vehicles was applied to investigate the temporal evolution of the mud pool geometry, revealing correlations between the pool and both meteoric rainfall and local hydrothermal volcanic activity^8^.

However, throughout the previous decade, resistivity imaging was likely the most widely adopted technique for collecting structural information about the entire central CF^18–20^ and the Solfatara crater^5,21–23^. For example, resistivity parameters were definitively capable of discriminating among the fluid circulation regimes in the Solfatara volcano-geothermal systems (by providing evidence of two-phase flows, prevalence of condensate, and gas reservoirs), and conceptual models of the Solfatara active fumaroles were elaborated on the basis of resistivity imaging^24^.

Specifically, concerning the Pisciarelli site (Fig. 1c), dedicated resistivity surveying was first reported through the electrical investigations presented in ref.^7^, who detected the permeation of deep source fluids in the very shallow structures of Pisciarelli. In this study, a reconstruction of the interplay with meteoric waters was conducted by analyzing the results of a 100 m long electrical resistivity tomography (ERT) survey with a 2.5 m electrode spacing repeated regularly starting in January 2014 and ending in April 2015. More recently, ERT was performed in the area by realizing three complementary ERT surveys conducted with a wider electrode spacing (10 m) with the aim of detecting subsurface fault and fracture systems while focusing on their roles in the accumulation and migration of gases injected from a deeper level in the subsurface^9^. In particular, two main fault systems have been identified, indicated with F1a and F1b in Fig. 1c, which seem to play a relevant role in the Pisciarelli setting.

The present paper aims to define the very shallow structure of the Pisciarelli hydrothermal system (down to 20 m depth) in its area of maximum degassing. A new resistivity survey was conducted during the first half of 2020, yielding a 3D image of the 60 × 80 m^2^ area (black box in Fig. 1c), hereafter referred to as the Pisciarelli fumarolic field (PFF), which comprises the soffione and the mud pool. The electrical tomograms reconstructed for the PFF are analyzed while also considering the results of time-domain induced polarization (TDIP) measurements, which were acquired through the same electrode array. Additionally, self-potential (SP) and temperature (T) surveys were carried out to map the behavior of such parameters at the ground surface in the area. Following the recent literature, we show how this combination of geoelectric and induced polarization surveying further improves the diagnostic capability of electrical techniques applied in volcanic environments and enhances the obtained results. This combination of tomograms provides in-depth insights into the evolution of the ongoing hydrothermal processes, thereby permitting the mechanisms governing the shallow circulation of volcanic hydrothermal fluids within the PFF to be defined.

## Results of the surveys

### The ERT model

The ERT data were utilized to reconstruct a 3D model of the subsoil resistivity in the PFF down to a 20 m depth. This 3D resistivity model consists, in its central part, of a structured rectangular lattice composed of 178 × 123 × 45 elementary cells, each one having Δx = Δy = 1 m and Δz = 1.5 m, representing the investigated volume. The outer part of the lattice was completed by cells having a spacing that increases with geometric progression. The electrical resistivity remains uniform within each cell. In other words, the ERT model consists of a series of 985,230 elements $(x_{n} , \;y_{n} ,\; z_{n} , \;\rho_{n} )$, where the n-th quadruplet has the coordinates of the center of the n-th elementary cell and the electrical resistivity computed by tomography in this cell. The electrical resistivity values range between less than 1 Ω·m and several hundred Ω·m, and the resistivity is rather variable within the shallowest part of a heterogeneous active and degassing volcanic area. To explore the complex resistivity distribution and to extract all of the relevant information, the volume resolved by tomography was divided into three parts, namely, a high-resistivity volume (HRV), a medium-resistivity volume (MRV), and a low-resistivity volume (LRV), by using two cutoff values defined as $\rho_{L} = 4$ Ω·m and $\rho_{H} = 20$ Ω·m. The choice of considering homogeneous volumes rather than single anomalies relies on the need to draw attention to the overall tomographic characteristics rather than the local heterogeneity, which is particularly true for a complex and active environment such as the shallow part of a volcanic area. Regarding the choice of thresholds, we adopted the same values considered in^21^ for the nearby Solfatara crater. Essentially, we first note in the 3D ERT model of Pisciarelli a general low-to-medium resistivity context, essentially analogous to what was observed near Solfatara. Subsequently, we started with a visual inspection of the maps, taking into account also a volcanological sketch of the area based on surface observations. Then, we classified the resistivity quite similar to the one adopted for Solfatara, also relying on previously published results concerning similar environments (ref.^21^ and references therein).

**Figure 2:**
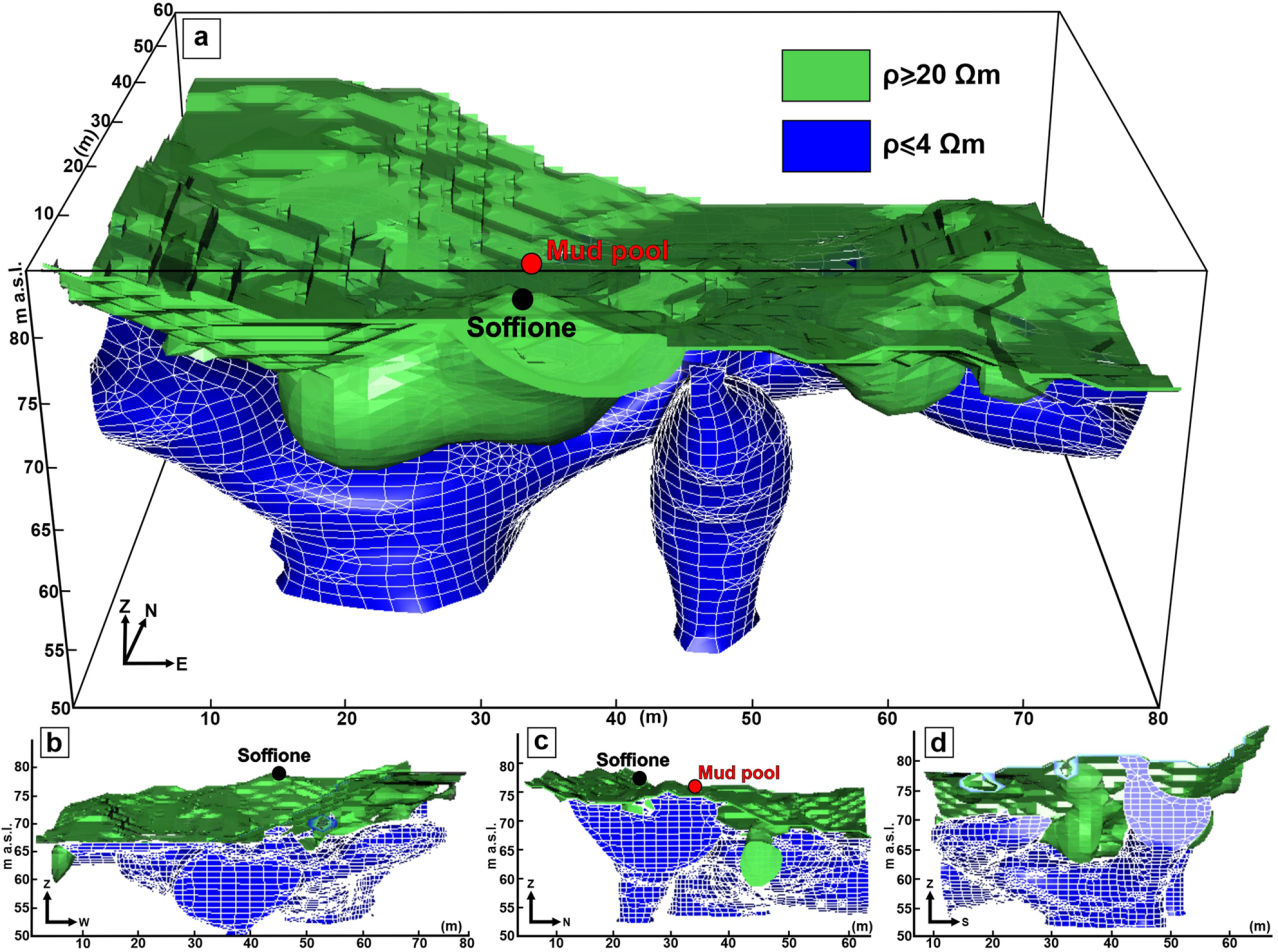
Sketches of the 3D resistivity model in four different views. The proposed visualizations are based on an isovolumetric representation of the electrical resistivity distribution reconstructed through the ERT survey. The volume resolved by tomography is divided into three parts: a high-resistivity volume (HRV, in green, where ρ ≥ 20 Ω·m), a medium-resistivity volume (MRV, omitted for a better graphical representation, where 4 Ω·m  < ρ < 20 Ω·m), and a low-resistivity volume (LRV, in blue, where ρ ≤ 4 Ω·m). The figure was created using the Visit 3.1.2 open-source software (https://wci.llnl.gov/simulation/computer-codes/visit) produced by the Lawrence Livermore National Laboratory (USA) and postprocessed using the Corel Draw 17 commercial software (https://www.coreldraw.com/it/product/coreldraw/).

The HRV and LRV are sketched in Fig. 2 (the MRV is omitted for a better graphical representation).

**Figure 3:**
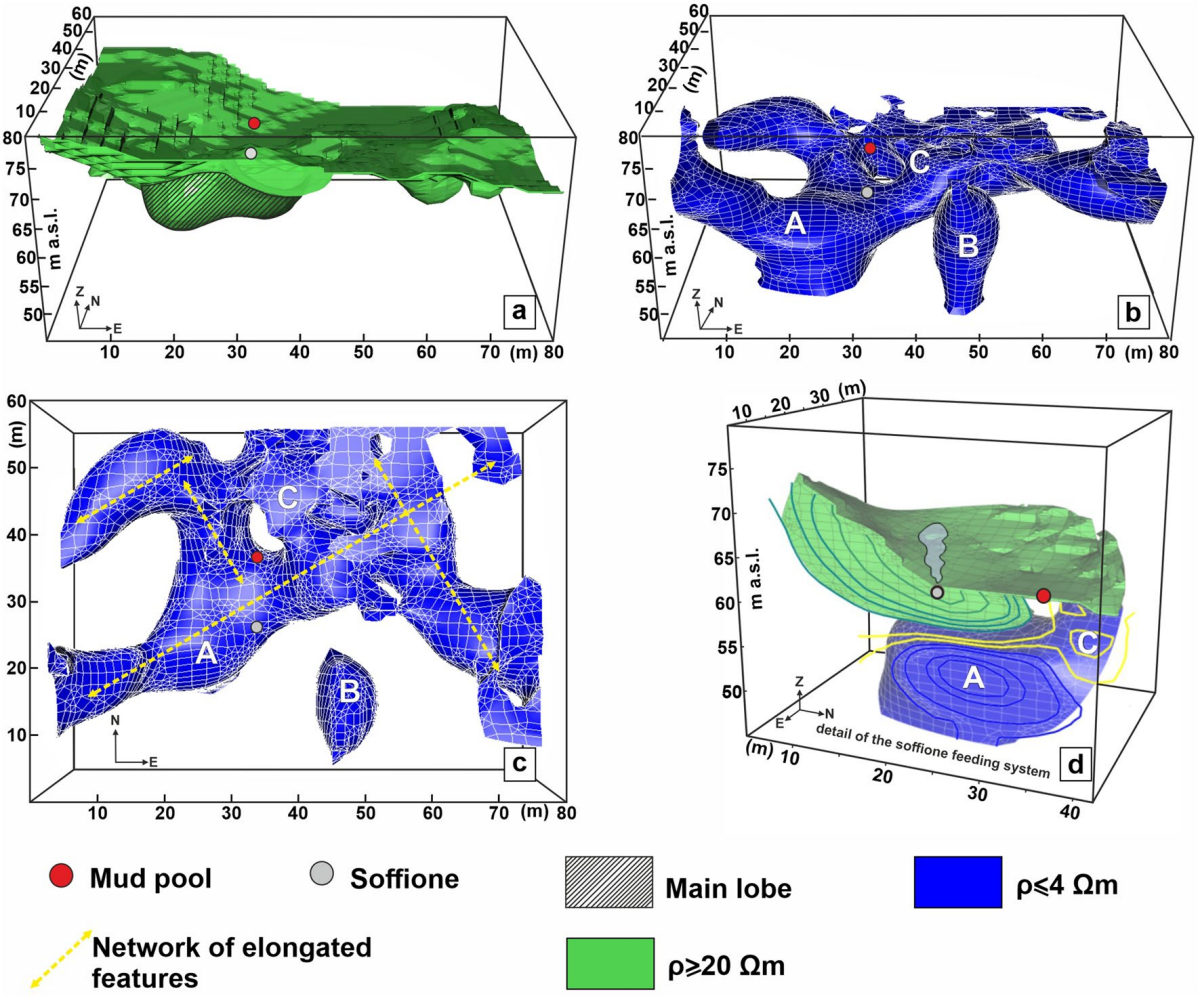
Details of the HRV and the LRV sketched in Fig. 2. The features discussed in the text are labeled with capital letters. The yellow dotted lines in panel (**c**) indicate the main alignments of the elongated branches that cross part of the LRV. The figure was created by the Visit 3.1.2 (https://wci.llnl.gov/simulation/computer-codes/visit) open-source software produced by the Lawrence Livermore National Laboratory (USA) and postprocessed using the Corel Draw 17 commercial software (https://www.coreldraw.com/it/product/coreldraw/).

As shown in Fig. 3a, the HRV could be associated with a sort of overburden deepening down the first 2–3 m below ground level (b.g.l.; see also the supplementary material), in addition to a region that extends deeper, down to a 5–6 m depth (hereafter referred to as the HRV main lobe).

As shown in Fig. 3b,c, the LRV seems to be characterized by a network of elongated anomalies developed along two mutually orthogonal directions, aligned N60°E and N30°W (yellow dotted lines in Fig. 3c) and coherently with the two main fault systems, F1a and F1b (Fig. 1c). Such features are confined to the resolved depths (the first 20 m of the subsoil). A prominent structure seems to rise from the depth and appears immediately below the HRV main lobe; this structure is depicted in Fig. 3c and is hereafter denoted with capital letter A. A second deep-rooted structure that is isolated and almost vertical appears in the SW sector of the LRV, as demonstrated in Fig. 3b,c with the capital letter B. Figure 3d shows a sketch illustrating the details of the soffione feeding system, where a slice crossing both the soffione and the mud pool extracted from the 3D resistivity model is presented as the contours. Also worth noting is that the mud pool corresponds to an MRV (indicated with the capital letter C in Fig. 3).

Additional 2D slices extracted from the 3D resistivity model are also provided in the supplementary material, further evidencing the structures previously described (adopting the same nomenclature).

### The TDIP model

**Figure 4:**
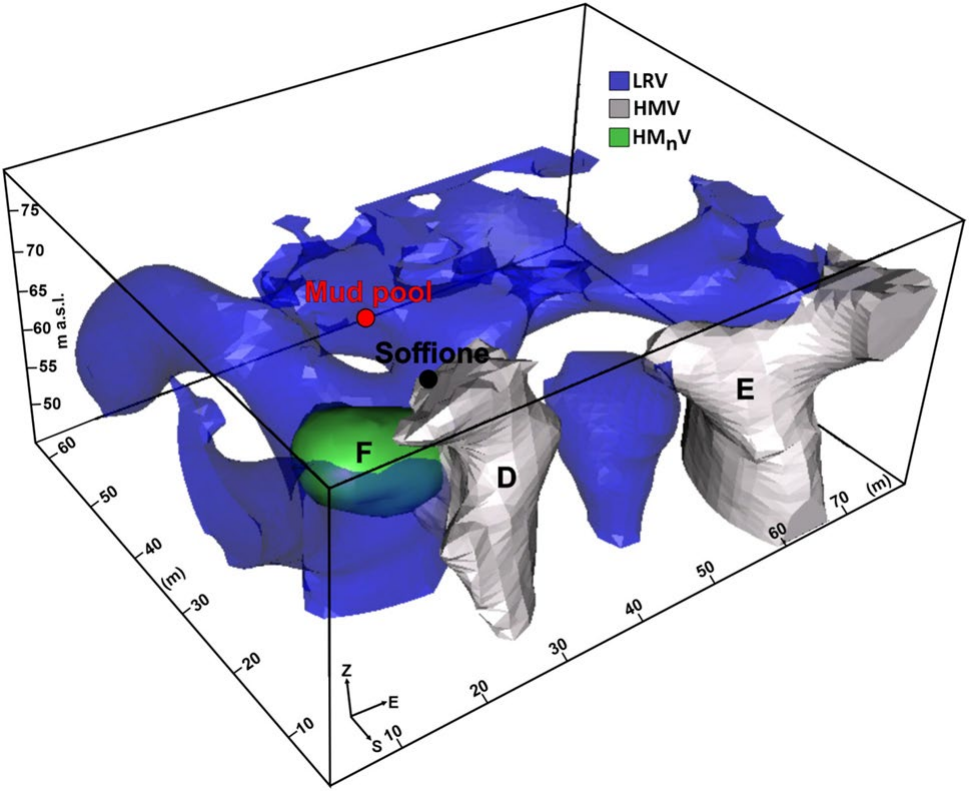
Sketch of the high-chargeability volume (HMV, in light gray) and the high normalized chargeability volume (HMnV, in light green) discussed in the text. Additionally, the LRV (see Fig. 3) is represented in blue. A threshold value of 10 mV/V was selected for electrical chargeability, whereas a cutoff value of 5 was selected for normalized chargeability. The figure was created by the Visit 3.1.2 open-source software (https://wci.llnl.gov/simulation/computer-codes/visit) produced by the Lawrence Livermore National Laboratory (USA) and postprocessed using the Corel Draw 17 commercial software (https://www.coreldraw.com/it/product/coreldraw/).

TDIP tomography yielded a 3D model of the electrical chargeability M, presented in Fig. 4, where the high electrical chargeability volume (HMV) is sketched in light gray (the LRV is sketched in blue for comparison). HMV was selected by extracting the cells of the TDIP model where the M value exceeds 10 mV/V, which is the third quartile of the chargeability series; this value was assumed to be the cutoff value to consider a structure polarizable. This HMV is essentially composed of the two spatially separated bodies (indicated in Fig. 4 and hereafter denoted with the capital letters D and E).

Figure 4 also shows the high normalized electrical chargeability volume (HM_n_V, in green), which was selected by adopting a cutoff value of 5 for the normalized chargeability. Normalized chargeability tomography detected the isolated body indicated in Fig. 4 (hereafter denoted with the capital letter F).

### The SP map

**Figure 5:**
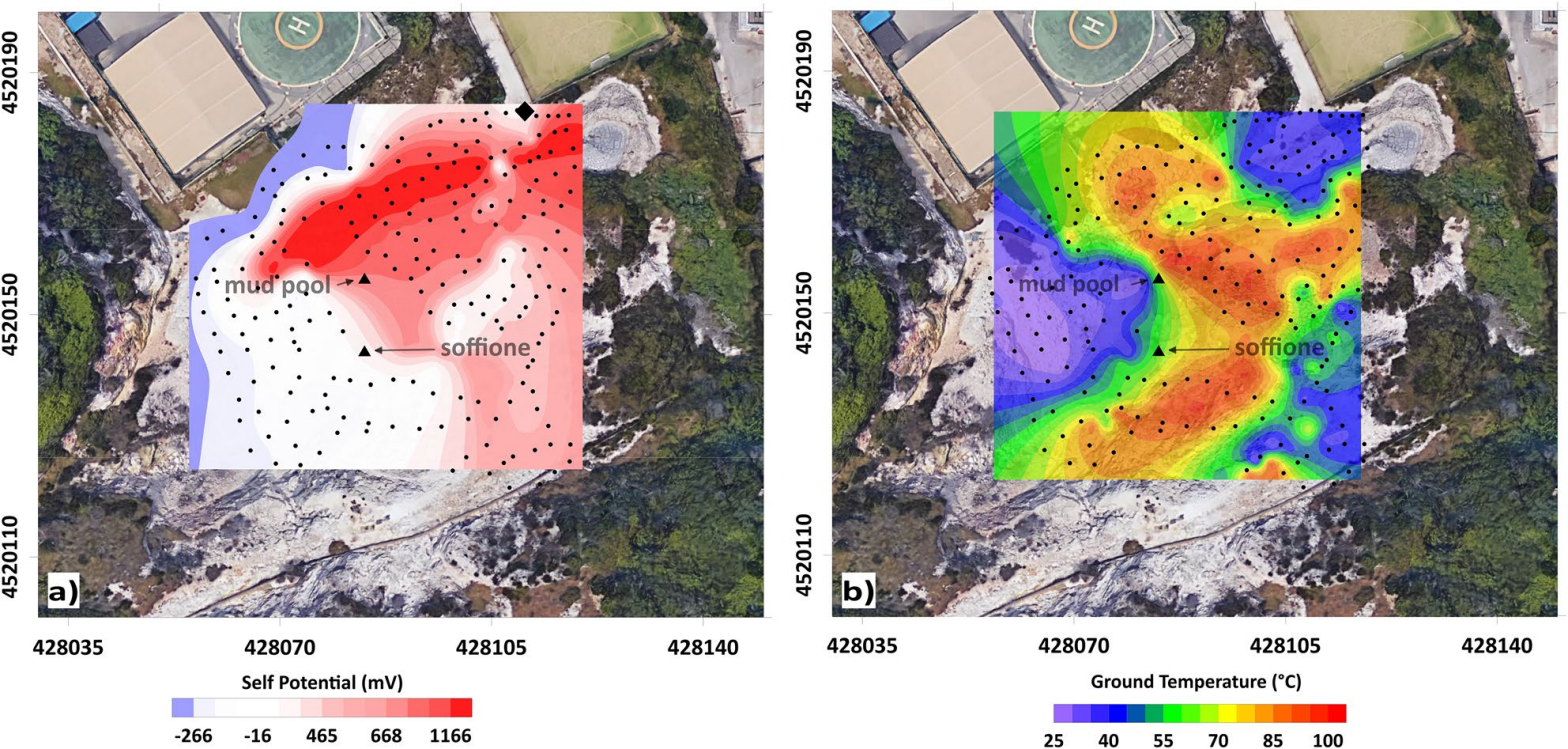
SP anomaly (**a**) and ground temperature (**b**) maps relative to the PFF. The color bars indicate the SP (in mV) and the ground temperature (°C) scales. In both maps, black dots indicate the measurement point positions. The black diamond in panel (**a**) indicates the zero-potential reference point. The figure was created using the MATLAB R2020b commercial software (https://www.mathworks.com/products/MATLAB.html) and postprocessed using the Corel Draw 17 commercial software (https://www.coreldraw.com/it/product/coreldraw/).

The processed SP data are represented in Fig. 5a in the form of a surface map.

The anomaly amplitudes vary from a few tens of millivolts up to 1600 mV. Such a high value is consistent with the values found in the literature^25,26^. In fact, on volcanoes, the observations of SP anomalies show a wide range of amplitudes, varying from − 2.7 V on the Adagdak volcano in Alaska^27^ up to + 1.8 V on Piton de la Fournaise^28^. Furthermore, the map in Fig. 5a makes it possible to note that such a high peak-to-peak excursion of the SP signal appears related to an abrupt variation in the electric potential corresponding to the main fault system F1a. In particular, such a steep rise in the SP is a rather well-known behavior, explained by the electrokinetic processes from the fluids flowing out from the fault^29,30^. Such an SP anomaly pattern suggests a separation of the PFF hydrothermal field into two contiguous sectors, likely originating from the effects of the main fault systems that govern fluid circulation.

A mapping of the ground temperature was also performed in the area using a K-thermocouple for temperature measurements at a 20 m depth. The map includes approximately 200 data points (black points in Fig. 5b) with a spacing of 3 m.

## Discussion

Electrical resistivity is highly sensitive to both clay-rich rocks and hydrothermal fluids. In volcanic environments, highly conductive zones generally correspond to regions in which clay coexists with hot water with temperatures below 200 °C^26^. However, the surface conductivity in active volcanoes contributes significantly to the overall electrical conductivity and cannot be neglected because it is necessary to quantify its influence on the resistivity results to avoid possible misinterpretation of ERT tomograms. Such an issue has been accurately debated in the literature (see, for example, ref., ^31–33^). As explained in the methodological section, electrical conductivity depends on a bulk contribution associated with conduction in the pore network and a surface contribution associated with the conduction path in the electrical double layer. However, the normalized chargeability that can be estimated during the TDIP investigation in field measurements (as M_n_ = M·σ) is directly related to the surface conductivity^32^. Consequently, in a volcanic environment, the integration of ERT and TDIP models offers a way to strengthen the results and avoid possible ambiguity in their interpretations. In effect, observing low conductivity and normalized chargeability can be considered typical markers of poorly altered volcanic rocks. Formations characterized by higher conductivity and normalized chargeability are typically composed of extensively altered rocks likely associated with clays.

An integrated approach for the interpretation of the 3D ERT (Fig. 3) and TDIP (Fig. 4) models is presented here, taking into account comparisons among the resistivity, chargeability, and normalized chargeability. The interpretation of resistivity data can also rely on the information that can be obtained considering the SP mapping on the surface. These models will be subsequently discussed together with the SP map (Fig. 5).

### The SP mapping and the PFF structural setting

**Figure 6:**
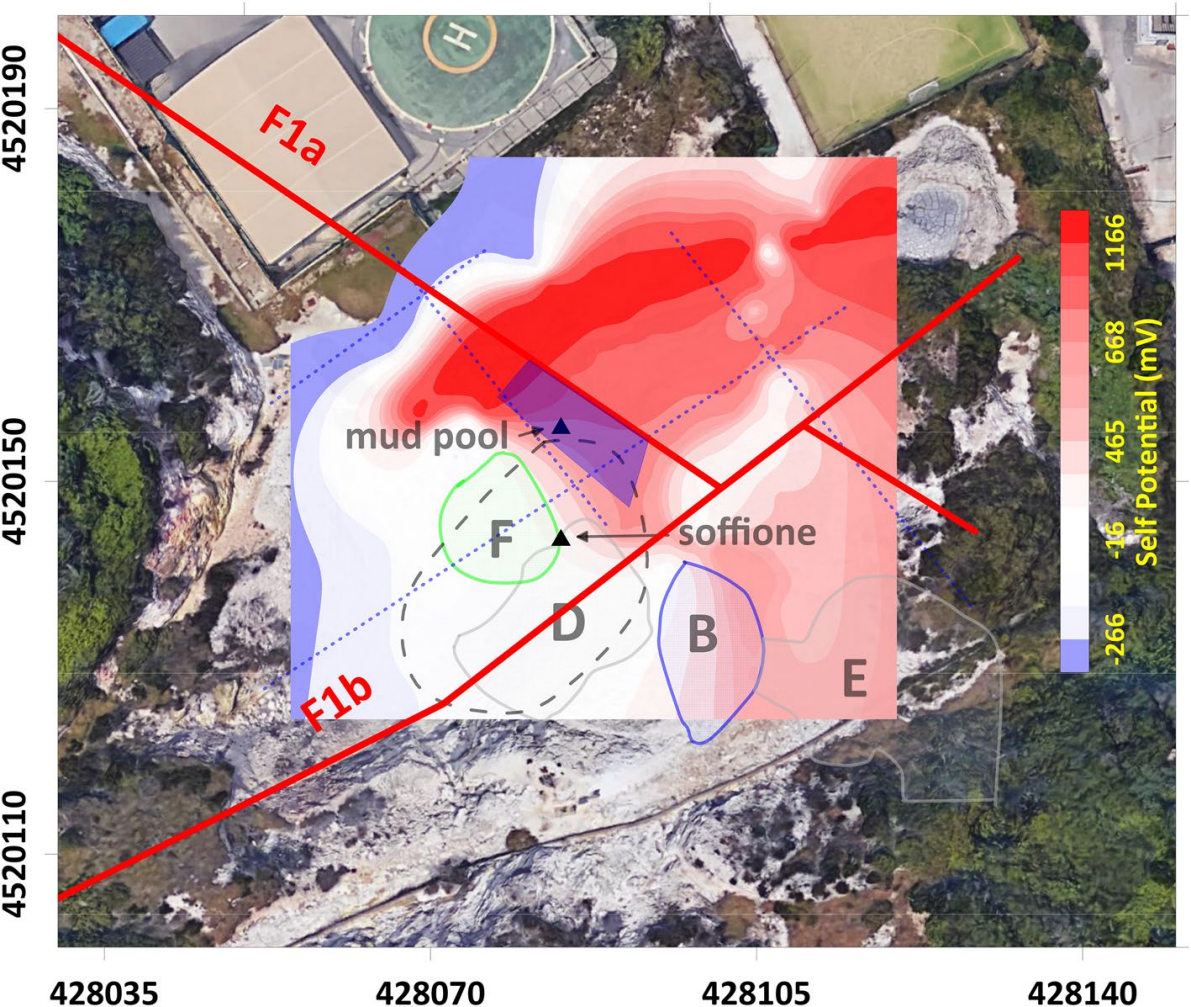
Traces of the main anomalies detected through the ERT and TDIP surveys (labeled with capital letters) superimposed onto the SP anomaly map shown in Fig. 5a. The color bar indicates the SP anomaly scale (in mV). The red lines indicate the two main fault systems crossing the area (redrawn from^9^). The blue dotted lines indicate the main alignments of the elongated branches that cross part of the LRV. The figure was created using the MATLAB R2020b commercial software (https://www.mathworks.com/products/MATLAB.html) and postprocessed using the Corel Draw 17 commercial software (https://www.coreldraw.com/it/product/coreldraw/).

Figure 6 shows the surface projection of the main anomalies previously described and superimposed onto the SP anomaly map. The map also displays the F1a and F1b fault lines identified in ref.^9^ through ERT surveying.

For a description of the nature of the main contribution to the SP anomaly, we refer to ref.^25^. The main contribution to SP on volcanoes relies on the electrokinetic contribution, which consists of large (up to several hundred mV) and positive anomalies because of the fluid flow through a porous medium that generates electric currents. Such an effect, often observed in active areas corresponding to fissure zones, is also likely to dominate in the case of the PFF, for which magmatic fluids and volcanic gases supply thermal energy that triggers convective thermal exchanges and, thus, massive hydrothermal circulations. This situation is ruled by the two major fault systems indicated in Fig. 6 (^9^ see also the discussion in section “The SP map”). In addition, SP anomalies can be sourced by electrochemical effects, which can be attributed to several phenomena, such as the diffusion of ions from a concentration gradient between two regions, the separation of the ionic charges occurring across a concentration gradient, and the so-called “membrane potential” linked to the presence of clayey materials, in which the counterions are attracted to the mineral surface while the co-ions are electrostatically blocked. In the case of Pisciarelli, and generally in the fumarolic areas, geochemical observations emphasize the existence of different ion concentrations in gas and water discharges^34^ and the likely presence of structures submitted to hydrothermal alteration and clay production^35^. Then, a chemical contribution to SP anomalies could be present. In contrast, A temperature-correlated effect involving the thermal conduction mechanism is rather unlikely because a significant thermal contribution to the SP anomaly requires the presence of thermal changes on the order of 500 °C in the field of superheated gas fluxes^25^.

As stated in section “The SP map”, based on the SP map, a division of the PFF can be introduced, namely, in the two NE and SW sectors. The NE sector of the PFF, marked by a highly positive spontaneous potential, is formed by rocks that scarcely tend to polarize, as indicated by the negligible values of M and Mn in the neighboring areas. The concomitance of high positive SP anomalies and low resistivity, chargeability, and normalized chargeability suggests strong electrolytic conduction into this volume, likely the effect of intense fluid circulation into porous rocks. Essentially, the NE sector corresponds to the part of the LRV where the network of elongated conductors is developed without being perturbed around the mud pool. The agreement between the alignment of these features and the directions of the F1a and F1b fault systems supports the proposed interpretation of a zone dominated by the circulation of hydrothermal fluids along fractures oriented favorably with respect to the symmetry of the main local structures coincident with the reduced injection of fluids from depth, as indicated by the fact that this part of the LRV appears to be confined to the first 10 m of the subsoil (Figs. 2 and 3; see also the supplementary materials). Conversely, in the SW sector of the PFF, this kind of symmetry is broken by the great majority of the electrical anomalies previously presented (the main lobe of the HRV, A, and B belonging to the LRV, D, and E belonging to the HMV, and F belonging to the HM_n_V), all of which are developed in this area. Moreover, all of the deep-rooted anomalies belonging to the LRV appear to be located in the SW sector of the PFF, supporting the idea of a possible influx of fluids from depth therein that complements the local hydrothermal circulation, generating a more complicated environment.

The separation of the SP anomaly map in a negative and a positive area, with a high correspondence to the fault trace—the base for the division of the PFF in the NE and SW sectors—is supported by the literature. As previously stated, the correspondence of the SP high with the F1a fault is based on the fact that faults and fracture systems favor the formation of permeable channels allowing fluid migration toward the surface, with the upwelling flow associated with the positive lobe of the SP. In the NE sector, interpreted as purely hydrogeological zones, the SP values are strongly controlled by the vertical distance between the measurement points and the water table, that is, the local thickness of the vadose zone^36^. In contrast, in the SW sector, the SP displays a minimum in the area of highest gas and heat fluxes, in the case of the existence of an additional flow system consisting of the downflow of condensate in the same area^37^. The coherence of this last observation with the interpretation of the nature of the SW sector of the PFF will be discussed in section “The basic mechanisms of the Pisciarelli hydrothermal system”.

### The PFF feeding system

**Figure 7:**
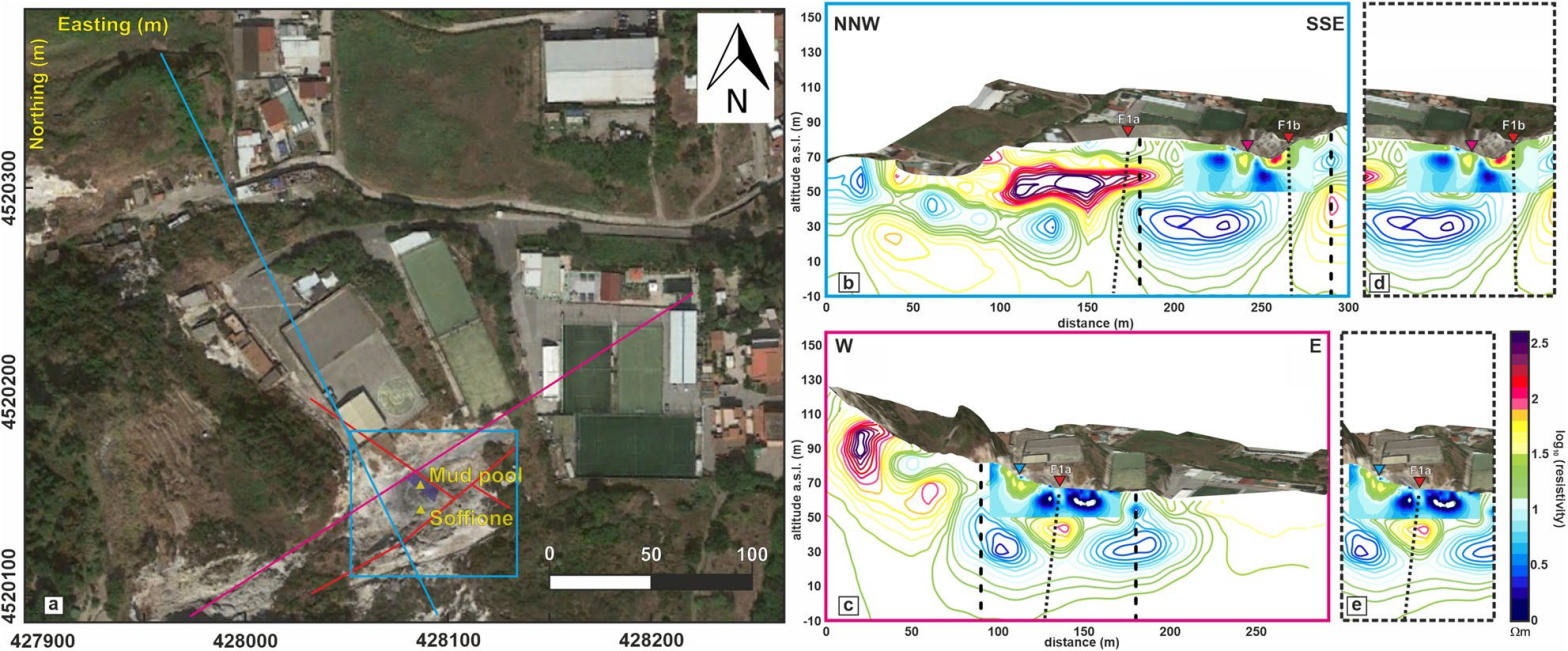
(**a**) Aerial view of Pisciarelli. The cyan and magenta solid lines indicate the alignments of two of the three geoelectrical profiles presented in ref.^9^. The cyan box encloses the area resolved by the present 3D high-resolution ERT. The red lines indicate the two main fault systems crossing the area (redrawn from ref.^9^). (**b**) 2D resistivity section relative to the cyan transect in panel (**a**), drawn as color contours. A 2D resistivity section extracted from the ERT model of Fig. 2 is superimposed as filled contours. The red triangles and black dotted lines indicate the F1a and F1b main fault systems detected in ref.^9^. The dotted black square encloses the part of the section magnified in panel (**d**). (**c**) 2D resistivity section relative to the magenta transect in panel (**a**), drawn as color contours. A 2D resistivity section extracted from the ERT model of Fig. 2 is superimposed as filled contours. The red triangles and black dotted lines indicate the F1a and F1b main fault systems detected in ref.^9^. The dotted black square encloses the part of the section magnified in panel (**e**). The figure was created using the MATLAB R2020b commercial software (https://www.mathworks.com/products/MATLAB.html) and postprocessed using the Corel Draw 17 commercial software (https://www.coreldraw.com/it/product/coreldraw/). The aerial view of Pisciarelli was produced by the Istituto Nazionale di Geofisica e Vulcanologia (INGV), Sezione di Napoli ‘Osservatorio Vesuviano’ (Curtesy of dr. Enrica Marotta).

Among the main anomalies previously described, conductive anomaly A is worth noting. This conductive feature rises to 5 m b.g.l. and presents a maximum lateral extent of approximately 15 m (at approximately a 10 m depth b.g.l.) (Fig. 3 and supplementary material). As previously stated, the Pisciarelli area was previously investigated by three ERT surveys, as reported in Isaia et al*.*^9^, two of which partly crossed the area investigated in the present study (Fig. 7a).

Figure 7b,c presents a comparison between the tomography results published in ref.^9^ and the slices extracted from the 3D ERT model of Fig. 3 in the corresponding directions. Figure 7d,e reproduce a magnified detail of the sections in Fig. 7b,c, respectively. In particular, Fig. 7b,d show how the A anomaly represents the offshoots of a larger conductive anomaly, interpreted in ref.^9^ as a highly fractured zone that allows shallow fluids to form a water/gas plume. Considering the TDIP model, the volume corresponding to conductive anomaly A presents low values of M and M_n_, suggesting that the A anomaly may represent the trace of a formation permeated by fluids that are in some way connected to a deeper reservoir. In contrast, the characteristics of the F anomaly indicate how the aforementioned summit of the channel corresponds to a conductive formation with high Mn and low M values. The relatively low chargeability indicates that the top part of the channel likely does not contain high quantities of mineral particles. However, the medium presents a strong tendency to polarize, as indicated by the high M_n_ values. The presence of a conductive structure maintaining a normalized chargeability signature despite a low chargeability could be interpreted as the trace of a clay formation^38^ that extends from 4 m to approximately 11 m b.g.l.; this formation is approximately 15 m and 20 m wide in the EW and NS directions, respectively (Fig. 4 and supplementary material). The hypothesis of a clay cap, which formed on the top part of the A channel due to the alteration effects of hot fluids rising from a deeper communicating reservoir, seems compatible with the chemical-physical conditions of the PFF. The pH of water pools and soils is neutral to acid but higher than that of the adjacent Solfatara, and enrichment in pyrite, illite, and feldspar at Pisciarelli has already been detected^35^. The same study suggested the existence in the PFF of major alteration subzones related to the appearance and disappearance of some mineral phases in response to changing physical–chemical boundary conditions mainly associated with weather circumstances.

It is useful to remark how two electrical studies considering structures affected by recent phreatic eruptions—White Island (New Zealand) investigated by ref.^39^ and Hakone (Japan), which is the subject of the study presented in ref.^40^—both indicate the presence of an extensive conductor that has been detected through ERT beneath the respective fumarolized areas. Importantly, we note the similarity of the shapes of the deeper reservoir identified at Pisciarelli through the ERT presented in ref.^9^ and the C4 conductor detected in the White Island (New Zealand) ERT by ref.^39^. The latter has been described as a permeability-bound layer containing single-phase liquid, an interpretation very similar to the suggestion previously presented of a deeper reservoir in which fluids in the liquid phase have been trapped because of the structural discontinuity represented by fault system F1a. Moreover, ref.^39^ also considered that temperature changes could allow the formation of mineral seals that create the permeability boundary trapping this liquid, a rationale similar to the idea of the formation of a clay cap in the summit part of the fluid rising channel A (Fig. 3). Finally, ref.^39^ emphasized a change in the depth of the C4 conductor, which does not coincide with the geomorphic crater boundaries, suggesting that lithological, permeability, and saturation boundaries do not follow geomorphic boundaries.

### The basic mechanisms of the Pisciarelli hydrothermal system

**Figure 8:**
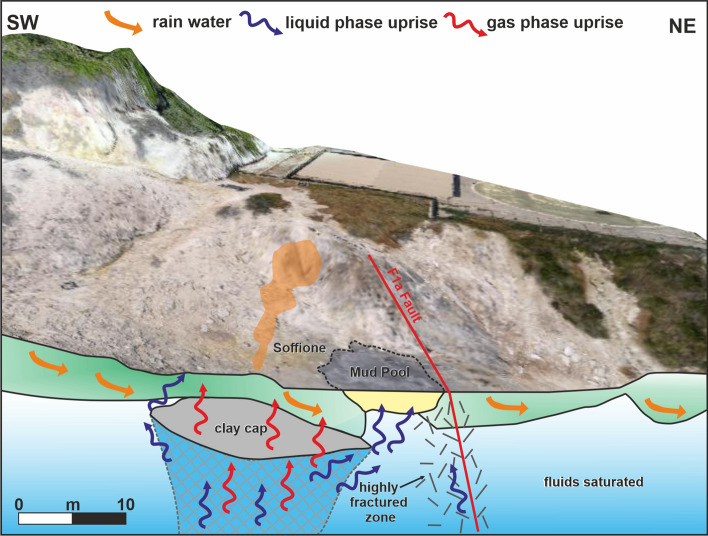
Structural setting of the PFF feeding system. The figure was created using the Visit 3.1.2 open-source software (https://wci.llnl.gov/simulation/computer-codes/visit) produced by the Lawrence Livermore National Laboratory (USA) and postprocessed using the Corel Draw 17 commercial software (https://www.coreldraw.com/it/product/coreldraw/).

The present ERT and TDIP models integrate previously acquired knowledge from ERT surveying, thereby highlighting the details regarding the setting of the shallow PFF feeding system. The structural model of the PFF shallow feeding system sketched in Fig. 8 suggests a few relevant details about the development and behavior of its hydrothermal system. The F formation, located at the top of the A anomaly, and faults F1a and F1b seem to exert the main control on the shallow fluid circulation pattern below the main fumaroles and the mud pool, even on the geometric variation of the latter. In particular, structure F1a acts as a permeability barrier for fluids, causing them to accumulate mainly in the deep reservoir and partly decreasing their northward migration (barrier effect). As this phenomenon continues, an overpressure develops in the deep reservoir, which induces the upwelling of fluids into the shallow PFF hydrothermal system. The fractures caused by the resulting increase in pore fluid pressure generate a channel whose electrical trace is conductive anomaly A, representing the conduit through which the fluids enter the shallow PFF hydrothermal system. Because these fluids completely ascend to the top of the channel, they encounter the HRV, which represents a highly compact and resistive layer likely corresponding to the Astroni deposits^9^. As a consequence of this additional permeability barrier, a sort of overpressure transfer occurs between the deep reservoir and the top of the channel where hot and overpressurized fluids rise from buoyancy accumulate, causing the extremely conductive anomaly localized at the top of the channel (overpressure transfer effect). This phenomenon induces an increase in both temperature and pressure at the top of the channel, in turn creating an environment that favors hydrothermal alteration of the host rock. This alteration could be the effect responsible for the generation of the clay formation corresponding to anomaly F located at the summit of the fluid upwelling channel corresponding to anomaly A. The proposed interpretation of the basic mechanisms of the PFF has some similarities with the phenomena that govern other fumarolic environments, probably related to the similarities of the physical conditions. Interestingly, an example is the resistive zone detected at Hakone (Japan), which intercalates the conductive layer localized beneath the fumarole area^40^. Such an anomaly has been interpreted as a vapor pocket based on the results of numerical simulations^41^ that revealed how an excess pressure zone in hydrothermal systems is formed immediately beneath a low permeability layer, and a steaming area is formed at the surface immediately above the excess pressure zone. Such a framework reproduces the setting proposed in Fig. 3d for the soffione feeding system, as indicated by the excess pressure zone that coincides with the area in which the clay cap formed and the HRV main lobe that represents the steaming area.

Finally, our interpretation of the PFF structural setting sketched in Fig. 8 suggests that the clay cap whose genesis has been previously described could play a relevant role in setting the local hydrothermal field by controlling the input of fluids into the shallow PFF. Experimental observations on fault rocks indicated that the permeability of water in clay-rich rocks tends to decrease as the effective pressure increases and remains generally lower than the permeability of gas^42^. Therefore, we hypothesize that the detected clay formation could govern the liquid influx into the shallow PFF. With increasing fluid pressure from an increase in the deep reservoir, the liquid phase should stop migrating when reaching the clay cap, acting as a permeability barrier. This clay cap could then hinder the passage of liquid-phase fluids through the shallow part of the Pisciarelli hydrothermal system, resulting in the local accumulation of mass and a consequent pressure increase. At the same time, gas transport would continue, potentially resulting in a sort of separation of the two phases (filtering effect) and a relatively major influx of gaseous fluid into the shallow part of the hydrothermal system. Such gas-rich fluids rising through the conduit would interact with fluids of meteoric origin, creating a two-phase mixing zone corresponding to the HRV main lobe. Recalling the interpretation in Gresse et al*.*^23^ of the resistivity anomalies in the adjacent Solfatara complex, we further hypothesize that the boundary of the HRV main lobe could mark the condensation surface of the gaseous phase. In the outside of this area, fluids in the liquid phase prevail. We also recall that this last consideration justifies the assessment of SP behavior in the SW sector of the PFF in “The SP mapping and the PFF structural setting”.

### The soffione and the mud pool

**Figure 9:**
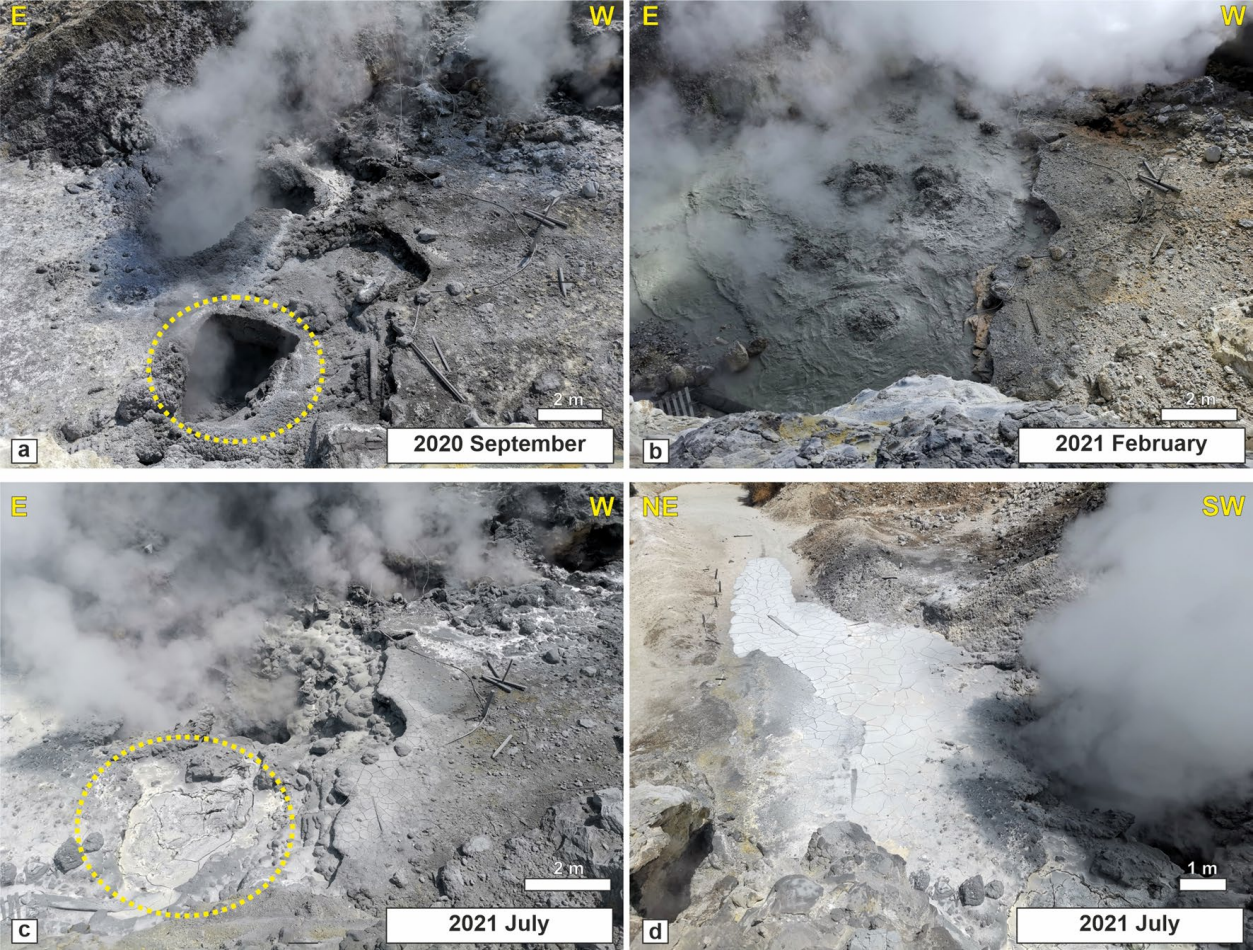
Photographic representation of the mud pool area in Sept 2020 (**a**), Feb 2021 (**b**), and July 2021 (**c**,**d**). Arrangement of pictures and photos by Roberto Isaia.

The mechanism described in section “The basic mechanisms of the Pisciarelli hydrothermal system” could help explain one of the as-of-yet unresolved issues. As previously mentioned earlier,  ref.^15^ assessed that the soffione releases > 600 tons/day of CO_2_, equivalent to the daily discharge of a medium-size arc volcano approaching an eruptive phase. However, despite the continuous morphological changes in the area and the vigorous but highly localized emissive activity, no signs of direct activation have been recorded. The filtering effect, which would alter the proportion between liquids and gases/vapors in the PFF, could be responsible for the extremely high volume of gas accumulation in the very shallow PFF hydrothermal treatment. The same mechanism could also help interpret the dynamics of the area with all of the variations within the PFF beginning in 2005, when new fumarole vents and a mud pool formed. In particular, we suggest that the detected shallow conduit, anomaly A, channels fluids toward the surface below the main fumaroles and that the clay cap on top of A facilitates the separation of the liquid phase, whose circulation is forced mainly within a narrow area confined toward the NE by fault F1a, where the mud pool is emplaced. The fluids induced toward the mud pool interact with rainwaters, also allowing the argillification processes discussed in section “The PFF feeding system”. In addition, given the general trend in the increase in gas flux and the structural control exerted by the F1a and F1b main faults, an area enlargement of the mud pool is favored exclusively within the SW sector. Therefore, the mud pool is the surface structure showing the most prominent change in morphology and dimension throughout the entire sector hosting the PFF (Fig. 9a,b), which has been modifying its physical/chemical characteristics for decades with the major variation in the emission area recorded in the last 15 years^9^. The dynamics of the mud pool, in the framework of the proposed model of the PFF feeding system, seem to have become more pronounced since 2020, when—after the summer period of scarce rainfall—very rapid variations have been accompanied by mud sputtering^9^ and complete drying of the mud pool vents (Fig. 9a). These particularly pronounced episodes, accompanied by a sharp drop in the value of the seismic tremor signal at the nearby station^3^, have been recorded during the final phases of liquid drying in the pool, confirming that variations in the fluids/liquid pressure can trigger these impulsive emission phenomena. A similar event repeated in 2021 led to the sealing of one of the main pool vents, which is now empty because of mud (Fig. 9a–c) and the generation of a mudflow with a length of approximately 20 m (Fig. 9d), suggesting that the contribution of muddy material from the northwest area likely corresponds to the clay cap first defined here (Fig. 8).

These episodes, partly linked to seasonal meteoric changes, together with the continuous recorded changes in the mud pool and fumarole configuration, can be explained mainly as the result of the enhanced phenomena within this shallow sector of the PFF through the basic mechanisms previously described. Taking into account these elements, modeling the proposed processes could help formulate evolution scenarios for the area and develop an appropriate strategy for better monitoring these structures.

## Conclusions

The region of Pisciarelli has exhibited a further increase in seismic activity and considerable morphological changes since 2020. As a consequence, access to the area, which was already prohibited to the public during the previous year, has been further restricted, and scientific activities are being carefully regulated because of the significance of an active and potentially hazardous area. The results of the ERT and TDIP surveys presented herein can be considered a relevant step in defining the subsurface structure of Pisciarelli and understanding the corresponding phenomena. The findings of new high-resolution ERT and TDIP surveys are integrated with the results of three ERT investigations previously performed in the area^9^, clarifying that these structures govern the fluid circulation pattern in the very shallow PFF hydrothermal system. At the same time, this newly acquired knowledge concerning the Pisciarelli structural setting allows a conceptual model of the fluid circulation in the shallow part of the PFF to be derived. This model indicates that the main fault systems serve as barriers to fluids circulating into the Pisciarelli hydrothermal system. Under the insulating effects of these faults, fluids first accumulate in a deep reservoir. Subsequently, as their pressure increases, their displacement is induced through upwelling along a channel, as detected by the high-resolution ERT survey, which acts as a conduit for fluid influx into the shallow PFF hydrothermal system. Moreover, this fluid dynamic favors the hydrothermal alteration of the top part of the upwelling channel and, thus, a second volume of fluid accumulates from the transfer of overpressure between the deep reservoir and the floor of a less permeable layer overhead, the bottom of which halts the ascension of hot fluids from the deep reservoir and causes them to accumulate. The induced change in the thermodynamic conditions of this volume favors the hydrothermal alteration of the host rock, triggering the formation of a clay structure that, in turn, affects the response to the variation in pore fluid pressure at the exit of liquids and gases from the channel, generating a sort of “filtering effect”.

This mechanism represents the first model proposed for fluid circulation within the PFF, part of the Pisciarelli area in which the most significant natural phenomena, such as the soffione and mud pool, are concentrated. This could help explain the abundance of the gases and vapors emitted by the main Pisciarelli soffione, which can be linked to the geometry and rock characteristics of the shallow sector of the fumarole fields. New geophysical images of the deeper part of this caldera sector, as well as investigations of the possible variations in the highlighted shallow structures of the PFF, could greatly elucidate the role played by the shallow fluid circulation system in the continuous increases in the fluid/gas supply from depth and, in turn, help better monitor the processes occurring throughout the PFF and evaluate the associated hazards.

## Method

### The complex conductivity model

Electrical conductivity describes the ability of a rock to conduct an electrical current, that is, a flow/flux of electrical charge. In contrast, chargeability describes the ability of a porous rock to reversibly store electrical charge during the passage of a primary electrical current^43^.

Models describing the low-frequency (${\text{f}} \le 100$ kHz) complex conductivity of a porous medium assume that charge transfer occurs via two parallel mechanisms^31^: (i) electrolytic conduction σ_el_, taking place in pore spaces, along grain boundaries, and within fractures and faults but negligibly through the silicate framework^44^ and (ii) surface conductivity σ_s_, which comprises all charge transfer phenomena, including the contributions of both conduction and polarization, associated with the electrical double layer (EDL) that exists at the interfaces of interconnected pore surfaces to counteract the net surface charge of the solid constituents^45,46^.

Because an electrolyte is essentially unpolarizable at low frequencies, electrolytic conduction can be described by a purely real term^18,23^, and it is possible to define the complex conductivity response of the rocks $\sigma^{*} = \sigma^{\prime} + i \cdot \sigma^{\prime\prime}$ as follows:

$$\sigma^{*} = \sigma_{el} + \sigma_{s}^{*} = (\sigma_{el} + \sigma_{s}^{{\prime}} (\omega )) + i \cdot \sigma_{s}^{{\prime\prime}} (\omega ).$$

We emphasize that the measurements acquired in the field $\left\lfloor {\sigma^{*} } \right\rfloor$ depend on both electrolytic and surface conduction, which causes the claimed ambiguity in the interpretation of conductivity^33,43,47^. We also note that considering the frequency dependence of the complex conductivity, the value of its real part is bounded by the value of the conductivity at zero frequency (σ_0_) and high frequency $(\sigma_{\infty } )$, whereas its imaginary part goes to zero under both conditions. The high-frequency conductivity $\sigma_{\infty }$ is the instantaneous conductivity of the material, that is, the conductivity experienced in Ohm’s law after the application of an electric field, whereas the DC conductivity σ_0_ is the conductivity of the material once all polarization phenomena have been fully established at all length scales^45^.

### Electrical resistivity tomography (ERT) and time-domain induced polarization (TDIP) tomography

**Figure 10:**
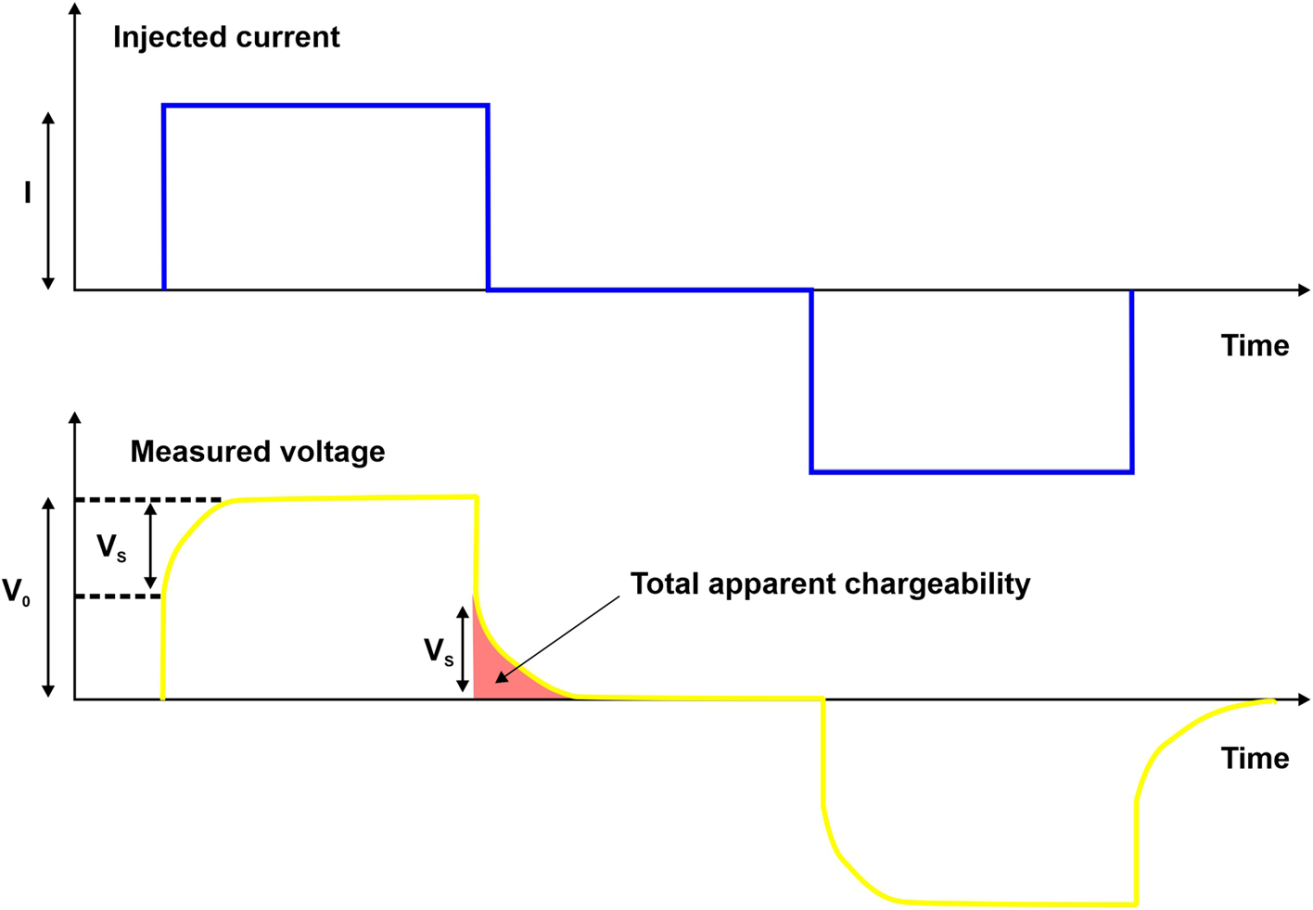
Representation of electrical measurements. The figure was created using the Corel Draw 17 commercial software (https://www.coreldraw.com/it/product/coreldraw/).

In the ERT method, a primary electric current *I* is injected into the ground between two source electrodes conventionally named A and B. This current induces an electric field and then a voltage drop V_0_ between two measurement voltage electrodes conventionally named M and N. The apparent resistivity $\rho_{a}$ is then estimated through the relationship $\rho_{a} = K\frac{{V_{0} }}{I}$ that relates $\rho_{a}$ [Ω·m] and the measured transfer resistance through the geometrical factor K, which depends on the relative positions of the electrodes. When the primary electric current is injected through the source electrodes, the material polarizes the stored charge and generates an electric field that decays with time, which opposes the flux of charge carriers into the material. As a consequence, the voltage difference between the measurement electrodes first builds up to a value Vs, called the secondary voltage, which reflects this effect (Fig. 10). This difference then progressively increases from V_s_ to the asymptotic V_0_ value. Then, when the primary current is shut off, the voltage suddenly decreases to V_s_ and subsequently decays progressively to zero^48^.

To remove background noise, a negative current is also injected (the − T_on_ period), where the sequence [+ T_on_, T_off_, − T_on_, T_off_] represents one TDIP measurement, in which the characteristics of the Vs decay curve are used to compute the apparent chargeability M while referring to the average intensity of the polarization phenomena between the electrodes. To accomplish this, the secondary voltage V_s_ is usually monitored through a set of integrated windows to obtain a better signal-to-noise ratio (SNR). For each window, the apparent chargeability is calculated using $M_{ai} = \frac{1}{{V_{0} (t_{i + 1} - t_{i} )}}\int_{{t_{i} }}^{{t_{i + 1} }} {V_{s} (t)dt}$, where M_ai_ [mV/V] is the apparent chargeability in window *i* between time t_i+1_ and time t_i_.

For the sake of completeness, although time-domain measurements are considered in the paper, we note that it is possible to define the induced polarization effect in the frequency domain. Accordingly, it is possible to introduce the Cole–Cole model^49^ for the complex conductivity as follows:

$$\sigma^{*} \left( \omega \right) = \sigma_{\infty } \left[ {1 - \frac{M}{{1 + \left( {i\omega \tau_{CC} } \right)^{c} }}} \right],$$

where *c* is the Cole–Cole exponent (dimensionless) and τ_CC_ denotes the Cole–Cole time constant [s]. In this definition, $\sigma_{\infty }$ again denotes high-frequency electrical conductivity^46,48^, whereas M denotes the intrinsic chargeability of the material, representing the same parameter previously introduced in the time domain. The term “intrinsic” indicates that M represents a physical property of a material.

It is possible to verify that $M\sim \frac{{\sigma^{\prime\prime}}}{\sigma ^{\prime}} = \frac{{\sigma_{S}^{{\prime\prime}} }}{{\sigma_{el} + \sigma_{s}^{{\prime}} (\omega )}}$, which highlights how M represents the polarization strength relative to the conduction strength in a porous material^31^. Recalling the aforementioned definitions of instantaneous conductivity and DC conductivity, worth noting is that M can also be introduced as the ratio $\frac{{\sigma_{\infty } - \sigma_{0} }}{{\sigma_{\infty } }}$.

This equation further indicates how M, which depends on both polarization and conduction, is not a direct measurement of the polarization strength. Hence, recalling that the polarization strength is 100–1000 times smaller than the conduction strength, it follows that $\left\lfloor {\sigma^{*} } \right\rfloor \cong \sigma^{\prime}$. It is possible to introduce a normalized chargeability, defined as $M_{n} = M \cdot \left\lfloor \sigma \right\rfloor \sim \sigma^{\prime\prime}$, which can be directly computed from the field measurements and can be considered the difference between the high-frequency conductivity and the DC conductivity. As discussed in ref.^45^, M_n_ is proportional to the cation exchange capacity of the material and, thus, to the clay content. This is not the case with M. However, in the presence of metallic particles (e.g., pyrite or magnetite), M is directly related to the volumetric content of those particles.

### Description of the ERT and TDIP surveys

To characterize the subsurface electrical properties of the PFF, eight profiles were deployed across the area (Fig. 1c). The measurements were realized using a Syscal Pro instrument (produced by IRIS Instruments) equipped with 120 acquisition channels. Source currents were supplied through the same Syscal Pro capable of injecting current up to 1 A. A dipole–dipole array was chosen because of its sensitivity to lateral changes in resistivity, greater depth of investigation, and quick acquisition time. Given both the harsh environment of the study area, which is characterized by vigorous emissions of high-temperature steam and gases, and the rugged topography, the electrodes were placed irregularly; the coordinates were stored using a handheld GPS with a 2 m precision. The survey was designed to combine lines of different lengths and different electrode spacings. Five profiles with a 3-m spacing and an approximate length of 70 m were realized contemporaneously: the profiles were deployed simultaneously in the field, and measurements were realized both in-line for each profile and cross-line between all acquisition lines, thereby acquiring a full 3D volume. Given the limited areal extent of the study area, which granted a good SNR level, measurements were realized considering the maximum possible source–receiver distance. Subsequently, three other profiles with a 1 m spacing and an approximate length of 25 m were singularly arranged to increase the density of the measurements around the soffione and mud pool. More than 9000 transfer resistances were recorded, each obtained by stacking five individual measurements; for the inversion, only the measurements with a standard deviation lower than 5% were retained. The data were subsequently filtered, eliminating the values exceeding the chosen threshold values. In addition, unrealistic apparent resistivities or excessively low voltage differences were discarded.

**Figure 11:**
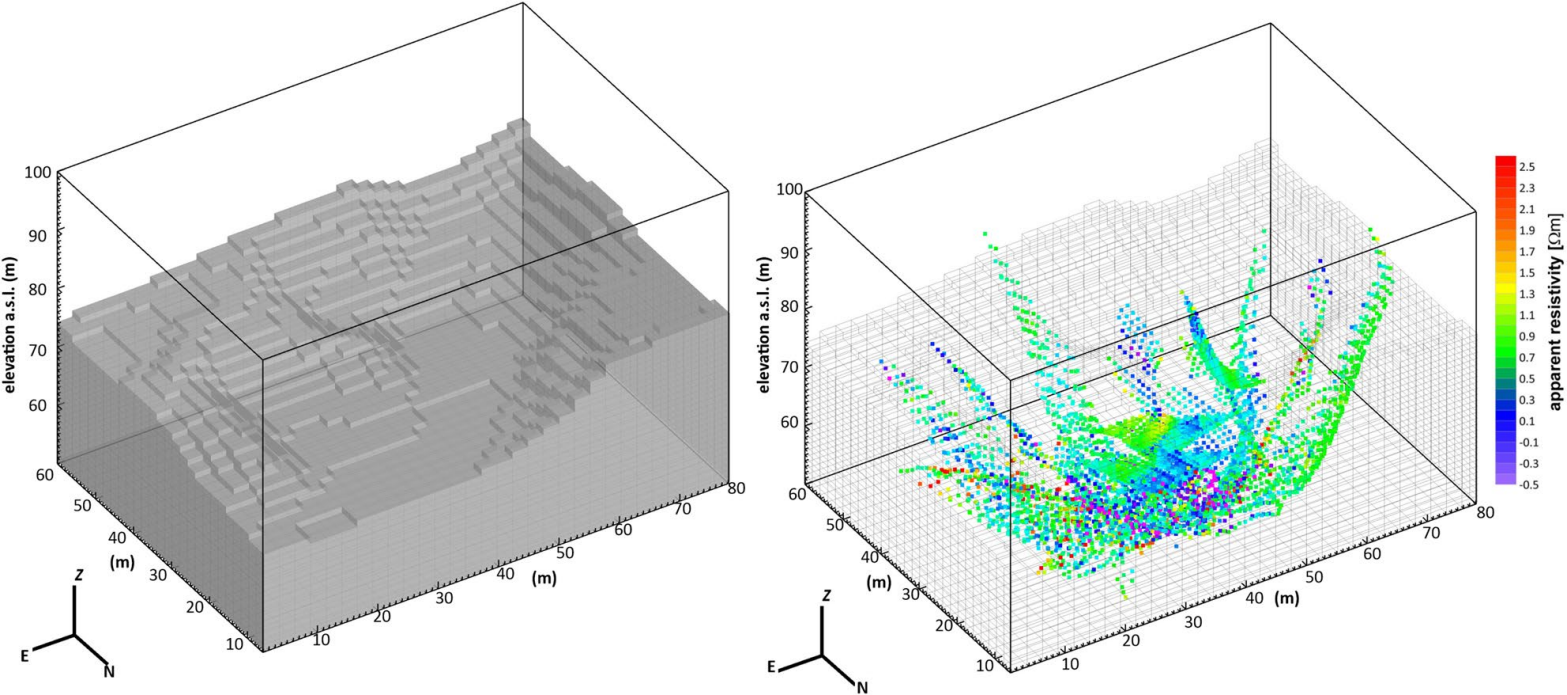
(**a**) Detail of the mesh adopted for the 3D inversion limited to the foreground of the discretized volume. The mesh is extended for kilometers for the sake of numerical convergence. (**b**) Common logarithm of the measured apparent resistivities (in Ω·m) represented as pseudopoints onto the 3D mesh. The figure was created by the Visit 3.1.2 open-source software (https://wci.llnl.gov/simulation/computer-codes/visit) produced by the Lawrence Livermore National Laboratory (USA) and postprocessed using the Corel Draw 17 commercial software (https://www.coreldraw.com/it/product/coreldraw/).

At the end of this filtering process, 6275 transfer resistances were retained for the inversion (the apparent resistivities in Fig. 11b), which was performed by adopting ERTLab Studio commercial software by Geostudi Astier s.r.l. (http://www.geostudiastier.it/area_en.asp?idCanale=56&sezione=1) based on the 3D inverse algorithm described by ref.^50^. An unstructured mesh comprising 178 × 123 × 45 elements and 985,230 nodes was constructed to represent the PFF (Fig. 11a). The mesh, which covered a rectangular area of ~ 4800 m^2^, was delimited by the realized electrical resistivity surveys. To ensure a common elevation baseline for all electrodes, the topographic elevations were recovered after implementing linear interpolation using a precise 1 m resolution digital elevation model (DTM) produced by processing a very high-resolution airborne LiDAR dataset acquired in 2009 by the Province of Naples Council in the framework of the CECOSCA Project^51^. The bottom of the mesh was set to 50 m b.g.l. based on the maximum depth of investigation reached by the longer ERT surveys. Finally, an external domain was created by extending the mesh both laterally and with depth to avoid boundary effects.

**Figure 12:**
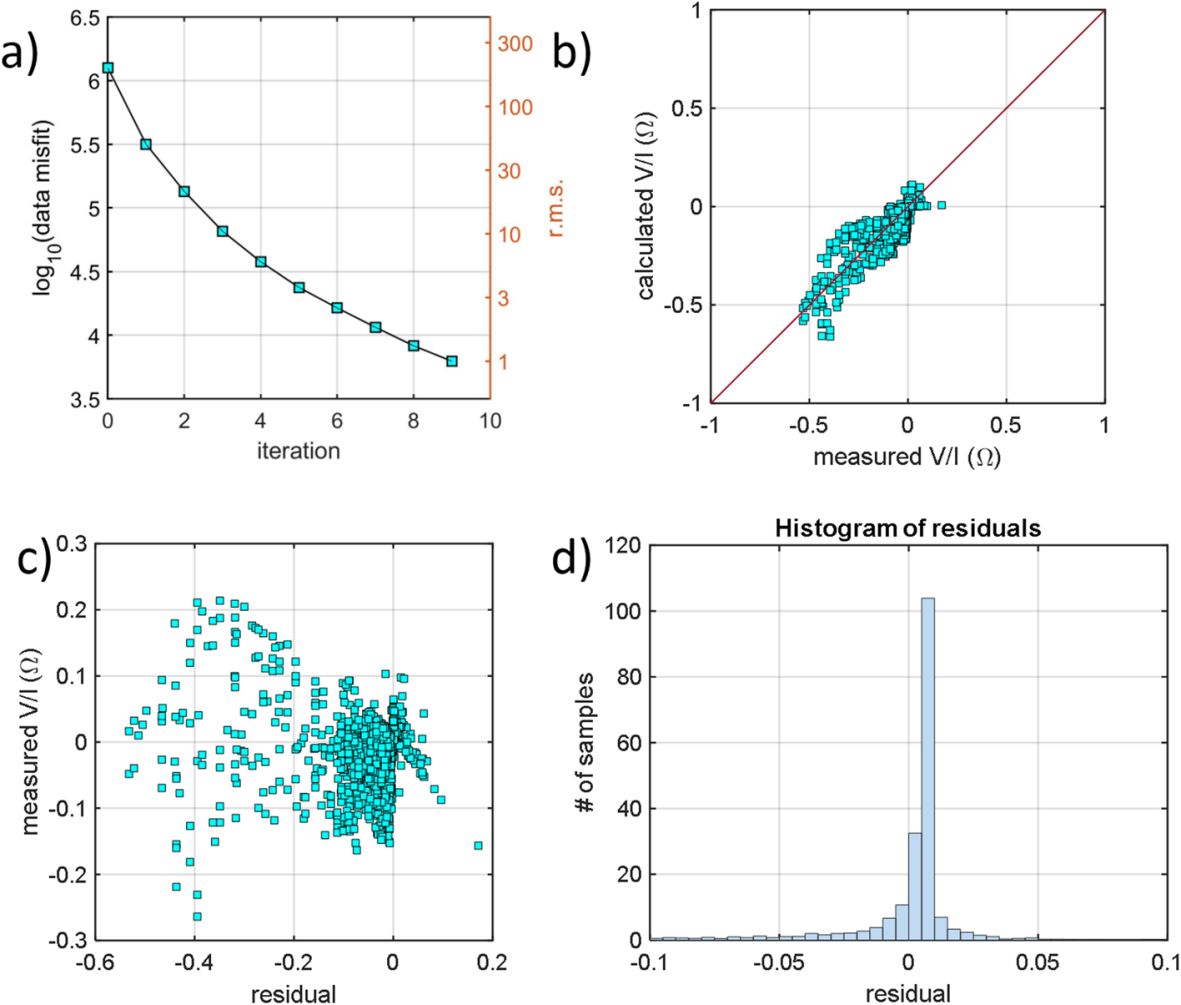
(**a**) Common logarithm of the data misfit plotted as a function of the number of inversion iterations. (**b**) Transfer resistances [Ω] calculated by the 3D inversion plotted as a function of the measured transfer resistances [Ω]. (**c**) Measured transfer resistances [Ω] plotted as a function of the residuals. (**d**) Histogram plot of the misfits. The figure was created using the MATLAB R2020b commercial software (https://www.mathworks.com/products/MATLAB.html).

Mixed boundary conditions were imposed at the mesh borders; namely, Neumann boundary conditions were applied at the ground surface, and Dirichlet boundary conditions were applied at the lateral and bottom boundaries of the model^52,53^. In addition, an initial model characterized by a homogeneous apparent resistivity of 10 Ω·m (the mean value of the measured apparent resistivity) was considered, and a 5% standard deviation for noise was assumed to invert the dataset. The inversion code finds a regularized solution when seeking the optimal value of the parameter vector **P** and the stabilization parameter α that minimizes the functional Y(**P**) = χ^2^ (**P**) + α**P**^**T**^**RP** and results in χ^2^(**P**) = χ^2^_prior_. The parameter vector **P** contained the natural logarithm of the conductivity of each mesh element, and **R**, the solution roughness, acted as the stabilizing functional. χ^2^_prior_ was equal to the number of data points, and χ^2^ was given by χ^2^ = **(D** − **F(P))**^**T**^**W (D** − **F(P))**, where **D** is the vector of known data values, **F(P)** is the forward solution, and **W** is a data weight matrix. The diagonal elements of **W** were the reciprocals of the data variances, and the off-diagonal elements were zero to assume noncorrelated data errors. The 3D inversion converged after 9 iterations with a root mean square error of 0.99. To assess the quality of the inversion, a few plots are presented in Fig. 12. In Fig. 12a, the common logarithm of the data misfit is plotted as a function of the number of inversion iterations. Figure 12b depicts the transfer resistances calculated by the inversion software as a function of the measured transfer functions. In Fig. 12c, the measured transfer resistances are plotted as a function of the residual, whereas Fig. 12d displays a histogram plot of the residuals.

To evaluate whether the 3D ERT model is resolved, the Jacobian or sensitivity matrix $S_{i,j} ({\text{m}}) = \frac{{\partial f_{i} (m)}}{\partial m}$, which contains the partial derivatives of the model responses with respect to the model parameters, has been estimated. ERTLab Studio provides a global sensitivity function equal to the diagonal of the matrix product S^T^S and that represents the sum (quadratic) of the sensitivities of all of the quadrupoles. The absolute values of the sensitivity function may vary over a wide range. Therefore, following ref.^54^, we computed a normalized sensitivity function, setting to 100 the maximum value and considering 3% of the normalized sensitivity function as a good indicator for the maximum resolution depth of our resistivity model. To this end, we show in the supplementary materials the corresponding sensitivity isosurface. In conclusion, the main electrical features discussed and interpreted in this work are well resolved.

### Self-potential anomalies

The self-potential (SP) method consists of measuring the potential difference between different points on the ground surface. Discussing the origins of spontaneous potential, ref.^25^ describe three different contributions. The first contribution is an electrochemical contribution, generally related to the chemical diffusion between ions of different concentrations within the soil that might generate anomalies because the ions themselves carry electric currents. In addition, oxidation–reduction processes can generate SP anomalies, typically in the presence of ore bodies. The second contribution is a thermoelectric contribution that is related to the appearance of an electric field through the rocks in the presence of a temperature gradient. The third contribution is electrokinetic or streaming potential, which is related to the electric potential gradient generated along the flow path by the motion of a fluid through a porous medium from the interaction between the moving pore fluid and the electric double layer at the pore surface. The SP survey was carried out by conducting measurements using nonpolarizable Cu/CuSO_4_ electrodes and a high-impedance voltmeter. Potential drops were acquired across a passive dipole with regular spacing of 3 m (black dots in Fig. 5a) moving along the interconnected circuits covering the area. During the survey, the initial point was removed, and the reference electrode was transplanted at the position of the last measurement using a leap-frog approach^55^. We also controlled the contact between the electrodes through the ground by checking the electric resistance before each measurement point. Thereafter, the entire dataset was corrected for the reference point (black diamond in Fig. 5a), which was assumed to have zero electric potential following the method proposed by ref.^56^. The reference for SP measurements is usually taken at the sea or a water table. Given that no such stable reference is available in the study area, the reference electrode was placed in a location without any hydrothermal manifestations at the surface. Subsequently, a closure correction was applied to reduce the effect of measurement drift as a consequence of possible changing conditions during the survey. The SP and T data were processed through the commercial software Surfer, produced by Golden Software, using the point kriging geostatistical method to produce an interpolated grid^57^.

## Supplementary Information

Supplementary Information.
