# Supplementary material for: The Pisciarelli main fumarole mechanisms reconstructed by electrical resistivity and induced polarization imaging
Source: Sci Rep. 2021 Sep 20;11:18639. doi: 10.1038/s41598-021-97413-1 (PMC8452721; doi:10.1038/s41598-021-97413-1)
Supplement: Supplementary file 1 — Supplementary Information. [file 41598_2021_97413_MOESM1_ESM.docx]

*Scientific Reports*

Supporting Information for

**The Pisciarelli main fumarole mechanisms reconstructed by electrical resistivity and induced polarization imaging.**

A. Troiano^(1,*)^, R. Isaia^(1)^, F. D. A. Tramparulo^(1)^, M. G. Di Giuseppe^(1)^

1 Istituto Nazionale di Geofisica e Vulcanologia, Sezione di Napoli ‘Osservatorio Vesuviano’ – Italy.

**Contents of this file**

Files SF1.dat and SF2.zip

Figures SM1 to SM3

**Introduction**

This Supporting Information file contains details on the ERT model presented in section 2 and its resolution.

File SF1.zip contains two tables of conversion for the electrodes. The first one is relative to the lines with 3 m spacing, that were acquired simultaneously. The second one is relative to the three 1 m spaced lines.

In Figure S1 the panel c of Figure 1 is reproduced, specifying the number of order of any electrode placed in the field.

Fig. S2 shows three resistivity cross-sections extracted from the 3D ERT and TDIP models along the transects depicted on the aerial map of the PFF (solid colored lines). The meanings of the symbols used in the map are illustrated in the legend.

Fig. S3 depicts the result of the sensitivity analysis performed on the ERT model of Figure 2-4. (a) isosurface at 3% of maximum sensitivity. (b) maximum resolved depth b.g.l. (c) maximum resolved depth a.s.l. (d) maximum resolved depth a.s.l. with the projection on the ground surface of the pseudopoints shown in Fig. 11. The trace of the main electrical anomalies discussed in the text is also reported on the maps.


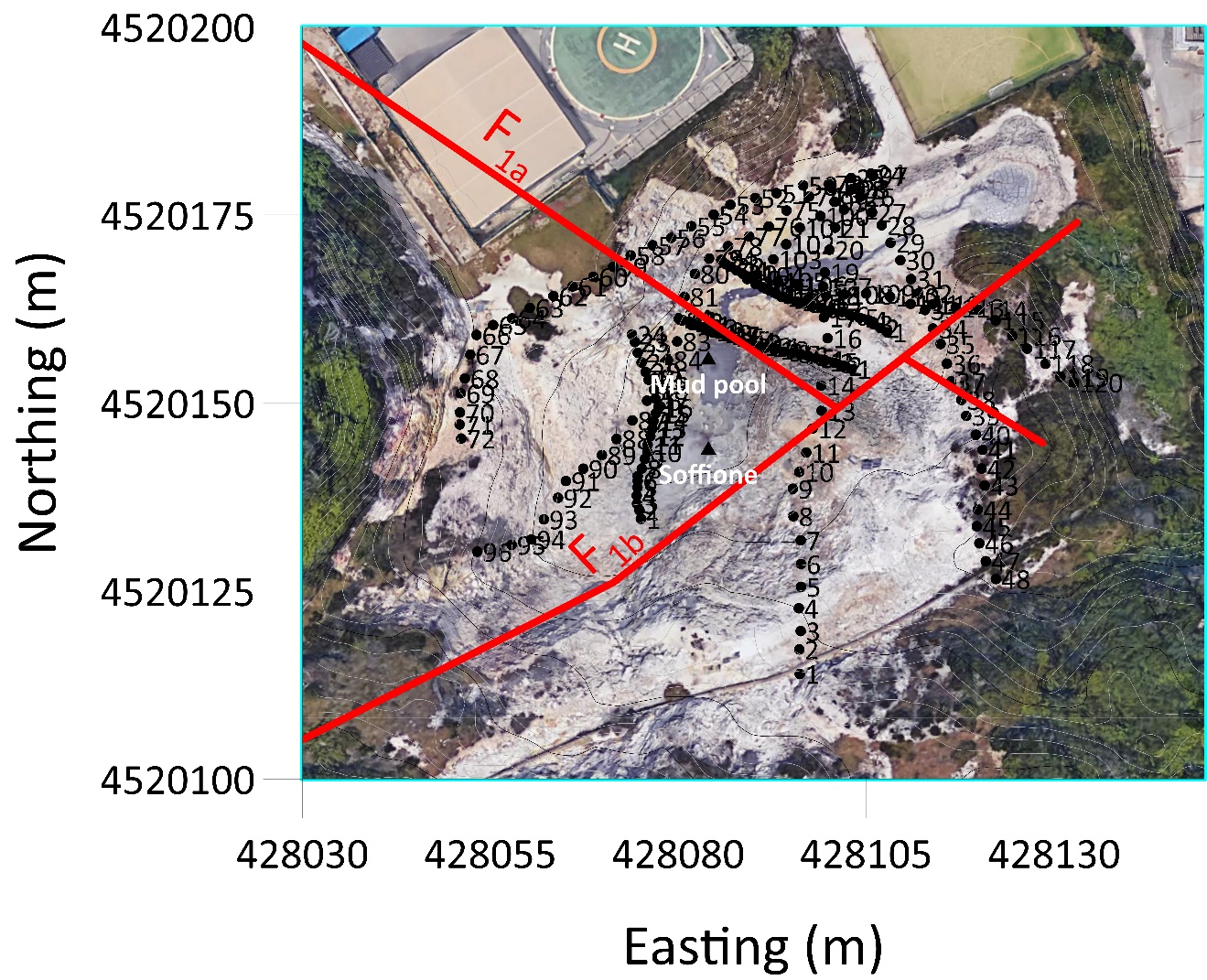


Figure S1.


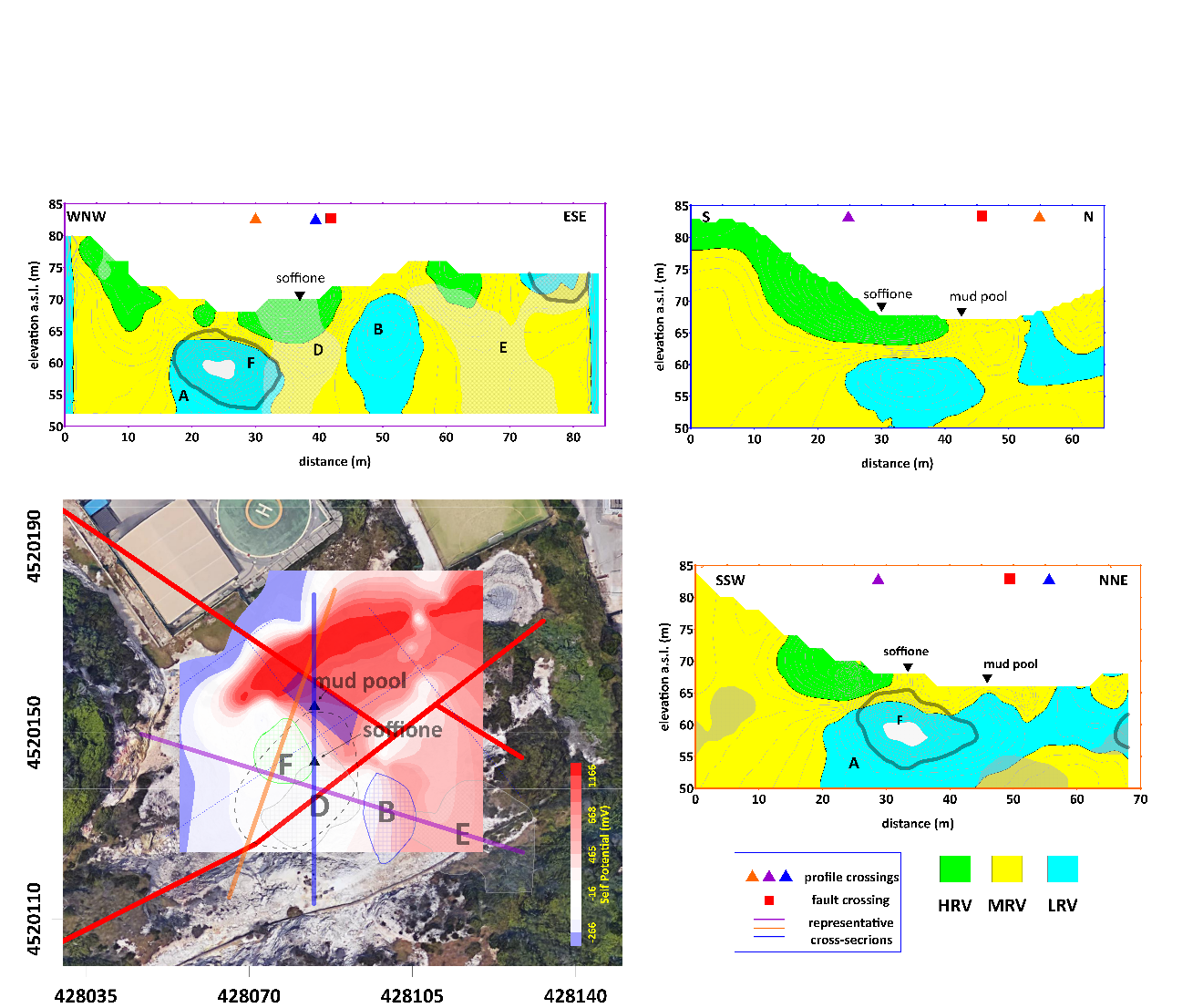


Figure S2.


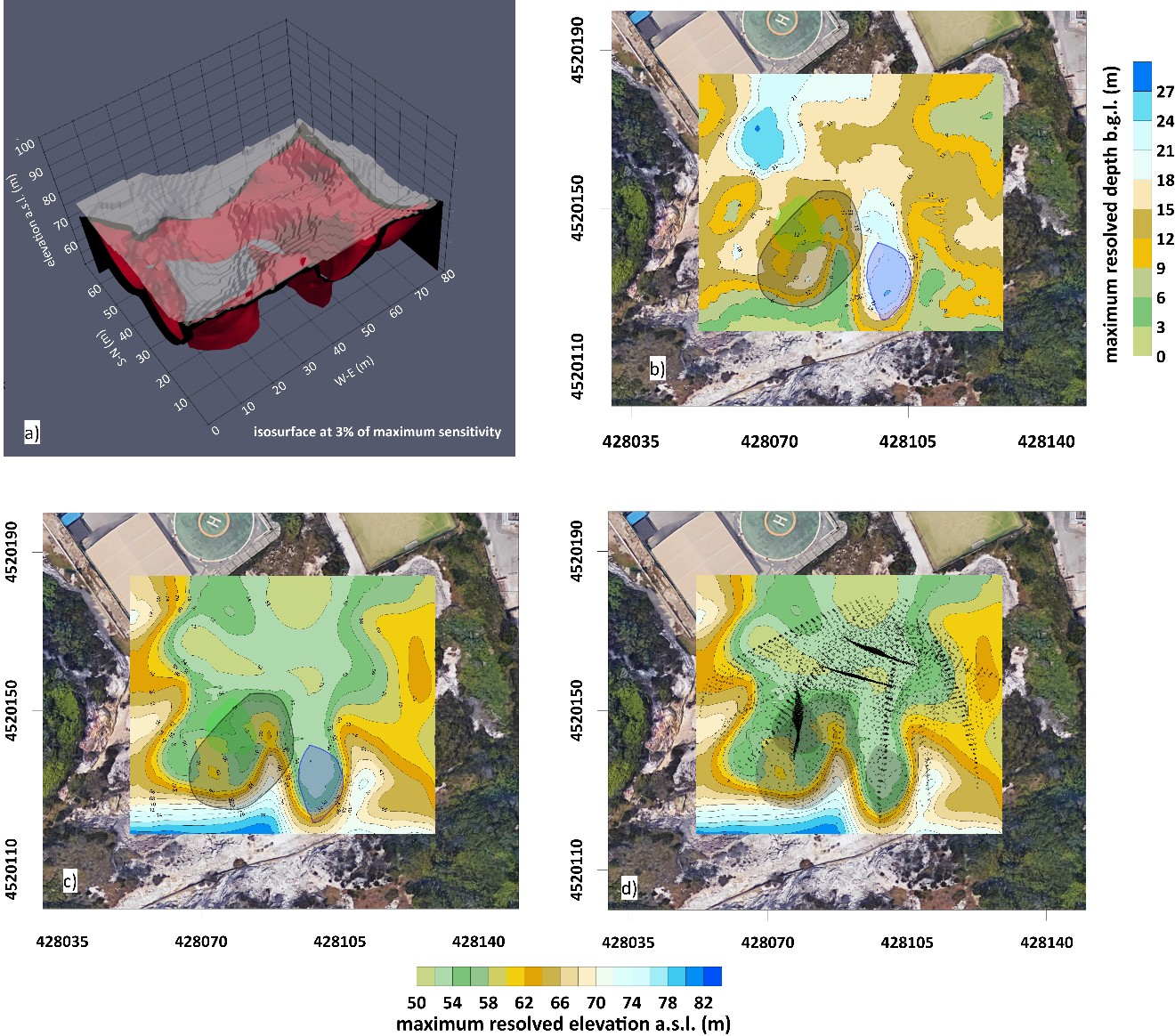


Figure S3.
